# Sequential Fractionation of Lignin for Interfacial Optimization and Enhanced Mechanical Performance in PBAT Composites

**DOI:** 10.3390/polym17172270

**Published:** 2025-08-22

**Authors:** Meng He, Mengfan Xu, Xian Yang, Chao Liu, Binghua Yan

**Affiliations:** College of Environment & Ecology, Hunan Agricultural University, Changsha 410128, China; hemeng202202@163.com (M.H.); 15274159798@163.com (M.X.); yangxian8760@163.com (X.Y.); binghuayan@hunau.edu.cn (B.Y.)

**Keywords:** lignin, fractionation, PBAT, biodegradable composite, interfacial compatibility

## Abstract

To address the inherent challenge of poor interfacial compatibility in lignin/poly(butylene adipate-co-terephthalate) (PBAT) composites, lignin was extracted from *Camellia oleifera* shells and subjected to sequential solvent fractionation using ethanol, acetone, and tetrahydrofuran (THF). Two representative fractions—acetone-soluble (ACL) and THF-soluble (THFL)—were selected for composite preparation with PBAT via solvent casting. The influence of lignin fractionation on the structural and performance characteristics of the resulting composites was systematically evaluated through Fourier-transform infrared (FTIR) spectroscopy, the water contact angle (WCA), differential scanning calorimetry (DSC), tensile testing, and scanning electron microscopy (SEM). The results revealed that the abundant hydroxyl groups and benzene rings present in both ACL and THFL facilitated hydrogen bonding and conjugation interactions with the PBAT matrix, significantly improving interfacial adhesion. Notably, the ACL fraction effectively suppressed phase separation and increased the glass transition temperature (*T_g_*) by 1.9 °C, leading to a 13.9% enhancement in tensile strength compared to neat PBAT. More strikingly, the incorporation of only 7 wt% THFL resulted in a remarkable 31% improvement in tensile strength. This substantial enhancement was primarily attributed to the favorable polarity match between THFL and PBAT, as well as the nucleating effect of THFL, which increased the crystallinity of PBAT by 25.3%. This study highlights the effectiveness of sequential lignin fractionation in tailoring the interfacial properties of biodegradable polymer composites. It also provides a promising strategy for the high-value utilization of lignin toward the development of high-performance, environmentally friendly materials.

## 1. Introduction

Poly (butylene adipate-co-terephthalate) (PBAT) is a recognized, promising biodegradable alternative to polyethylene, offering good flexibility and processability [1]. PBAT is extensively used in areas such as food packaging and agricultural films [2]. However, the high cost and relatively low mechanical strength of PBAT limit its application [3,4]. To address these limitations, PBAT is frequently blended with other polymers, including polylactic acid (PLA) [5], polycaprolactone (PCL) [6], starch [7], and lignin [8]. Among these, lignin, as the second most abundant natural organic polymer in plants after cellulose, is attractive due to its low cost, biodegradability, and inherent properties such as antioxidant activity and structural rigidity [9,10]. Consequently, modifying PBAT with lignin has emerged as a significant research focus [4,11,12,13]. Nevertheless, the substantial polarity difference between lignin and PBAT hinders the formation of a stable interface during direct blending, often leading to phase separation, which adversely affects the composite’s performance.

To improve the performance of lignin/PBAT composites, several methods have been developed to enhance interfacial compatibility, such as lignin nanosizing [14,15], chemical modification [16,17], the addition of compatibilizers [12], and lignin fractionation using solvents [4]. Among these approaches, solvent fractionation has attracted considerable attention due to its procedural simplicity and its ability to yield lignin fractions with improved homogeneity in composition and structure. This enhanced homogeneity is conducive to designing composites with desirable mechanical strength [18,19]. Although acetone is a commonly used solvent for lignin fractionation [20,21], research on fabricating composites by blending PBAT with lignin derived from multi-solvent fractionation remains limited. Furthermore, lignin/PBAT composites are typically prepared via melt blending, a process that requires high temperatures to melt both components. However, previous studies have found that compression and high temperatures reduce the content of functional groups (especially aromatic OH group) in lignin while increasing its average molecular weight and polydispersity index. Solvent casting offers a viable alternative, enabling the dissolution of lignin and PBAT at lower temperatures and under mild conditions [22]. This method, by preserving lignin’s structural integrity, thus holds significant research value and application potential.

The ethanol-soluble lignin fraction with the greatest polarity difference from PBAT was removed. Then, the acetone-soluble lignin fraction and the tetrahydrofuran-soluble lignin fraction were mixed with the tetrahydrofuran solution of PBAT, and lignin/PBAT composite film materials were prepared by the solvent casting method. This study aimed to investigate the improvement in the compatibility between different organic solvent lignin fractions and the PBAT matrix and explore the effects of the addition of lignin fractions on the thermal properties, mechanical properties, and wetting properties of the composite materials, with the expectation of providing technical references and data support for the development of low-cost and high-performance PBAT-based composite materials.

In this study, lignin extracted from *Camellia oleifera* shells using a deep eutectic solvent (DES) was sequentially fractionated with three different organic solvents: ethanol, acetone, and tetrahydrofuran (THF). The resulting acetone-soluble (ACL) and tetrahydrofuran-soluble (THFL) fractions were then individually blended with poly(butylene adipate-co-terephthalate) (PBAT) in a THF solution to fabricate lignin/PBAT composite films via the solvent casting method. The compatibility of these lignin fractions with the PBAT matrix was investigated. Furthermore, the effects of their incorporation on the thermal stability, mechanical performance, and wetting properties of the resulting composites were systematically evaluated. This work aims to provide a basis for developing low-cost, high-performance, bio-based PBAT composite materials.

## 2. Materials and Methods

### 2.1. Materials

**Figure 1:**
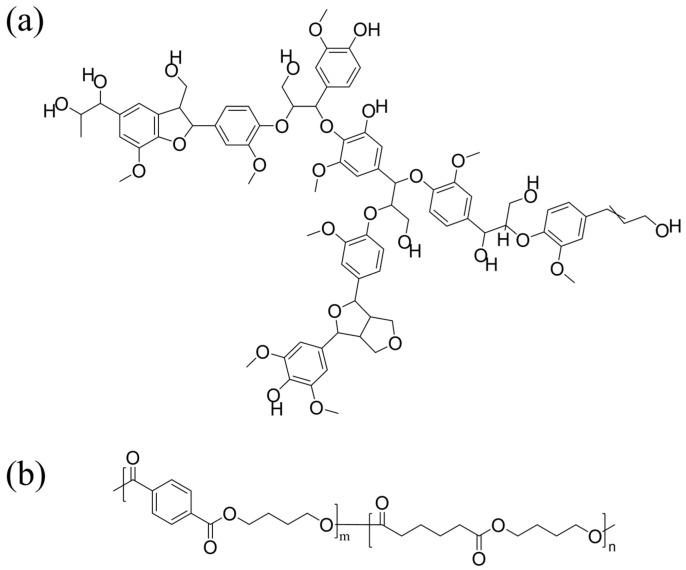
The chemical structure of lignin [23] (**a**) and PBAT (**b**).

Lignin (DL), from *Camellia oleifera* shells, was extracted using a deep eutectic solvent (DES) (Lactic Acid–Choline Chloride = 10:1) under the conditions of 120 °C, 6 h, and a liquid-to-solid ratio of 20 in the laboratory. PBAT (C1200) was from BASF (Ludwigshafen, Germany). The chemical structures of lignin and PBAT are shown in Figure 1. Anhydrous ethanol, acetone, and tetrahydrofuran, all analytical grade, were purchased from Sinopharm Chemical Reagent Co., Ltd. (Beijing, China). All chemicals purchased were used without any further purification.

### 2.2. Lignin Fractionation Using Organic Solvents

Lignin extracted from *Camellia oleifera* shells using DES (DL) was sequentially fractionated with three organic solvents: ethanol, acetone, and tetrahydrofuran (THF). The fractionation commenced by suspending 2.0 g of DL in 20 mL of ethanol, followed by stirring (500 rpm, 2 h) at room temperature (RT). The resulting suspension was then separated by centrifugation into a soluble supernatant and an insoluble residue. The residue was re-extracted twice under identical conditions, and the supernatants from all three cycles were combined. The combined supernatant was concentrated under reduced pressure (e.g., by rotary evaporation) to remove the ethanol. The resulting solid was dried in a vacuum oven at 35 °C to a constant weight to yield the ethanol-soluble fraction (ETL). The dried ethanol-insoluble residue was then subjected to the same fractionation procedure protocol, first using acetone and subsequently THF, to obtain the acetone-soluble fraction (ACL) and the tetrahydrofuran-soluble fraction (THFL), respectively. The final insoluble material was collected as the residue (RL). Each treatment was performed in triplicate. The yield of each fraction was calculated according to the following equation:

yield (%) = (mass of dried fraction/initial mass of DL) × 100%

For sustainability, solvents from each step were recovered via rotary evaporation for potential reuse.

### 2.3. Preparation of PBAT/Lignin Composite Film

PBAT/lignin composite films were prepared via a solvent casting method. Typically, 1.5 g of PBAT and a predetermined amount of DL were dissolved in 30 mL of tetrahydrofuran. The mixture was then heated in an oil bath at 65 °C for 1 h to ensure the PBAT was completely dissolved, yielding a homogeneous casting solution. Subsequently, the solution was cast onto a clean glass plate using a wet-film applicator (SZQ, LEIQI, Shanghai, China) to a wet thickness of 300 μm. The film was then allowed to dry at RT to facilitate solvent evaporation. Composite films were prepared with DL concentrations of 0, 1, 3, 5, 7, and 10 wt% relative to the PBAT mass and were designated as P, P/DL1, P/DL3, P/DL5, P/DL7, and P/DL10, respectively. ACL and THFL were added in the same way to prepare composite films, which were designated as P/ACL and P/THFL, respectively.

### 2.4. Characterization

#### 2.4.1. Fourier-Transform Infrared (FTIR) Spectroscopy

FTIR analysis (Spectrum 65, Perkin Elmer, Seer Green, UK) of raw lignin was carried out via the KBr pressing method. The composite film was tested with ATR-FTIR analysis (Spectrum 65, Perkin Elmer, Waltham, MA, USA), with air spectra used as the background. The wavenumber range was 4000–400 cm^−1^, the resolution was 4 cm^−1^, and the number of scans was 64.

#### 2.4.2. Molecular Weights

The molecular weight and its distribution of lignin were determined using a GPC system (E2695, Waters, Milford, MA, USA). Prior to analysis, the lignin was acetylated in a 1:1 (*v*/*v*) mixture of pyridine and acetic anhydride to enhance its solubility in the mobile phase, tetrahydrofuran. The resulting acetylated product was dissolved in tetrahydrofuran and filtered through a 0.22 μm filter. The GPC test was performed in THF solution at 35 °C with an elution rate of 1.0 mL min^−1^ on the Waters E2695 GPC system equipped with a refractive index detector. The separation was achieved using Agilent PLgel Mixed-B and PLgel Mixed-C columns connected in series. The system was calibrated with polystyrene standards.

#### 2.4.3. Water Contact Angle (WCA)

The contact angle of the films was measured using a contact angle goniometer (SL200B3, KINO, Los Angeles, CA, USA). The analysis was conducted using deionized water as a probe solution.

#### 2.4.4. Mechanical Properties

The tensile mechanical properties were evaluated using a tensile testing machine (ETM104B, WANCE, Shanghai, China). All films were cut into 50 mm × 10 mm strips to determine the mechanical properties. The tensile speed was set to 20 mm/min.

#### 2.4.5. Differential Scanning Calorimetry (DSC)

The thermal properties were analyzed using a differential scanning calorimeter (Q2000, TA Instruments, New Castle, CA, USA). To erase the thermal history, the samples were first heated from room temperature to 180 °C at a nitrogen flow rate of 50 mL min^−1^ for 5 min. Subsequently, the samples were cooled to −80 °C, held isothermally for 2 min, and then reheated to 220 °C. The heating and cooling rates throughout the entire test process were both 10 °C min^−1^, and the cooling curve and the secondary heating curve were recorded. The *T_g_* (glass transition temperature), *T_m_* (melting temperature), *T_c_* (crystallization temperature), Δ*H_m_* (enthalpy of fusion), and χ (crystallinity) of the films were obtained through the above process. The crystallinity of PBAT was calculated by χ $\left(\%\right)=\frac{{∆H}_{m}}{{∆H}_{\mathrm{PBAT}}^{0}\times X_{\mathrm{PBAT}}}\times 100$ [24], where *X*_PBAT_ represents the mass fraction of PBAT and ${∆H}_{\mathrm{PBAT}}^{0}$ is the theoretical enthalpy of melting of 100% crystalline PBAT (114 J·g^−1^).

#### 2.4.6. Scanning Electron Microscopy (SEM)

SEM (Sigma 300, Zeiss, Oberkochen, Germany) was carried out to analyze the surface and cross-sectional morphologies of the films. For cross-sectional analysis, the films were cryo-fractured in liquid nitrogen. All samples were mounted on conductive adhesive tape. Gold spraying was performed on the sample surface to improve the electrical conductivity of the surface.

## 3. Results and Discussion

### 3.1. Comparison of the Different Lignin Fractionations

**Figure 2:**
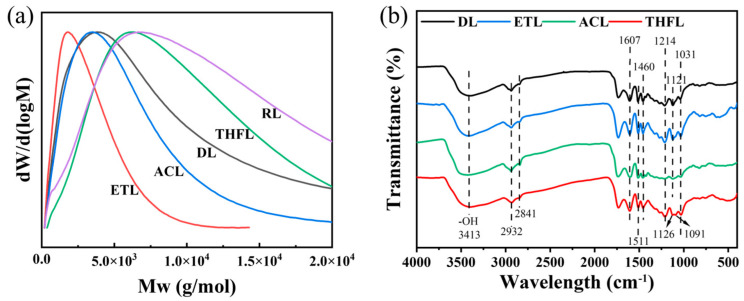
Molecular weight (**a**) and FTIR spectra of different fractions of lignin (**b**).

The dissolution behavior of lignin is known to vary significantly in different organic solvents due to differences in solvent polarity and hydrogen bonding interactions between lignin and solvents [25]. The yield and molecular weight data of organosolv-fractionated *Camellia oleifera* shell lignin are presented in Table 1. The sequential fractionation of *Camellia oleifera* shell lignin using ethanol, acetone, and tetrahydrofuran yielded 31.70%, 14.64%, and 19.77%, respectively. The relative molecular weights of the fractions increased in this order. ETL and ACL exhibited lower molecular weights than DL, while THFL showed a higher molecular weight than DL. Interestingly, RL possessed a higher weight-average molecular weight (*Mw*) but a lower number-average molecular weight (*Mn*) compared to THFL. This suggests that ethanol and acetone failed to completely extract the lower-molecular-weight lignin fractions, a portion of which remained in the RL. As evidenced in Figure 2a, THF demonstrated superior selectivity for lignin molecular weights. Furthermore, all fractionated lignin samples showed a reduced polydispersity index (PDI), indicating a narrower molecular weight distribution and improved homogeneity after fractionation.

The FTIR spectra of the DL and fractionated lignin are presented in Figure 2b, showing that the main structural features of the DL remained largely unchanged after sequential fractionation using the three organic solvents. A broad absorption band at 3413 cm^−1^, characteristic of both aliphatic and phenolic hydroxyl groups, was observed across all samples. This confirms the preservation of these functional groups, which are crucial for lignin’s solubility, interfacial properties, and mechanical performance [26,27]. The characteristic peaks at 2932 and 2841 cm^−1^ were attributed to C-H stretching vibrations from methyl and methylene groups, while the aromatic skeleton vibrations appearing at 1607, 1511, and 1460 cm^−1^ indicated the intact aromatic structures in all lignin fractions. The presence of phenolic hydroxyl groups was further confirmed by the C-O stretching vibration at 1214 cm^−1^, and the β-O-4 ether linkages were evidenced by the absorption peak at 1031 cm^−1^. Notably, the C-O stretching vibration of methoxyl groups in syringyl units (originally appearing as a single peak at 1121 cm^−1^) split into two distinct peaks at 1126 and 1091 cm^−1^ in the THFL. The methoxy vibration peak of the guaiacolic unit is typically around 1140 cm^−1^, but in low-molecular-weight lignin fractions, the guaiacolic unit is often mixed with the syringic unit [28]. THF enriches high-molecular-weight fragments, splitting the originally overlapping vibration peaks into two independent peaks. This may be due to an increase in the proportion of guaiacolic units in THFL.

The polarity differences between ethanol, acetone, tetrahydrofuran, and PBAT decrease sequentially, with ethanol exhibiting the greatest polarity mismatch with PBAT. Based on the variations in the molecular weight and functional groups of lignin fractions, combined with the principle of “like dissolves like”, the initial removal of ETL—the fraction with the most dissimilar properties to PBAT—would enhance the compatibility of subsequent lignin fractions with PBAT. Therefore, DL, ACL, and THFL were selected for preparing PBAT/lignin composite films.

### 3.2. FTIR Analysis of PBAT/Lignin Composite Films

**Figure 3:**
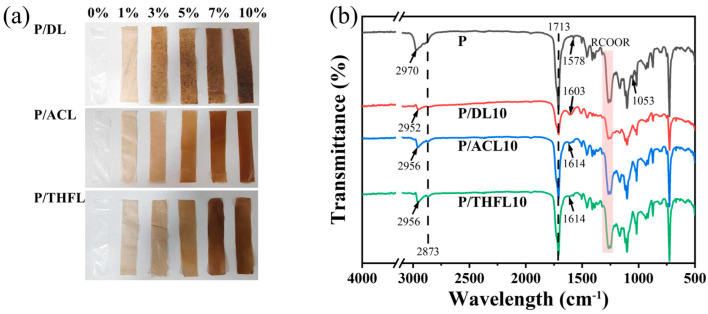
Visual appearance (**a**) and typical FTIR spectra of PBAT/lignin composite films (**b**).

PBAT films are typically transparent milky white [29], while the incorporated lignin is a dark brown powder. As shown in Figure 3a, with increasing lignin content, the color of the PBAT/lignin composite film gradually darkens from light brown to dark brown, and P/DL exhibits obvious lignin agglomeration. FTIR analysis was employed to investigate the interactions between lignin and PBAT. As shown in Figure 3b, the infrared spectra of PBAT, P/DL10, P/ACL10, and P/THFL10 were compared. The peaks at 1713 cm^−1^ and 1205–1302 cm^−1^ correspond to the C=O stretching vibration and C-O-C asymmetric stretching vibration of the PBAT ester group, respectively. The weakening of the intensity after the addition of lignin indicates that lignin has a shielding effect on the absorption peaks of the PBAT ester group. The sequential increase in ester group intensity in P/ACL and P/THFL indicates an improvement in lignin dispersion.

After the addition of lignin, the C-O absorption peak (1053 cm^−1^) of PBAT disappears, possibly due to the polar groups of lignin (such as hydroxyl groups) forming hydrogen bonds with the C-O bond, thereby masking its vibrational signal. The asymmetric stretching vibration (2970 cm^−1^) and symmetric stretching vibration (2873 cm^−1^) of the methylene group in PBAT are affected by the addition of lignin: the absorption peak at 2970 cm^−1^ shifts to lower wavenumbers, while the absorption peak at 2873 cm^−1^ broadens, attributed to the hydrogen bonding between the hydroxyl groups of lignin and the C-H bonds in the methylene groups of PBAT.

After adding DL to PBAT, the skeletal vibration of C=C on the aromatic ring of PBAT at 1578 cm^−1^ disappears, and the characteristic peak of the lignin benzene ring at 1603 cm^−1^ appears. This may be due to the π-π interaction between DL and the aromatic structure in PBAT, which suppresses the vibration of the aromatic ring in PBAT, while the vibration signal of DL’s own aromatic ring becomes dominant. After adding ACL and THFL, the intensity of the aromatic ring absorption peaks in PBAT significantly decreases, and a new absorption peak appears at 1614 cm^−1^. This indicates that the π-π interactions between lignin and the aromatic structures in PBAT are weakened after the addition of ACL and THFL.

### 3.3. Wetting Properties of PBAT/Lignin Composite Films

**Figure 4:**
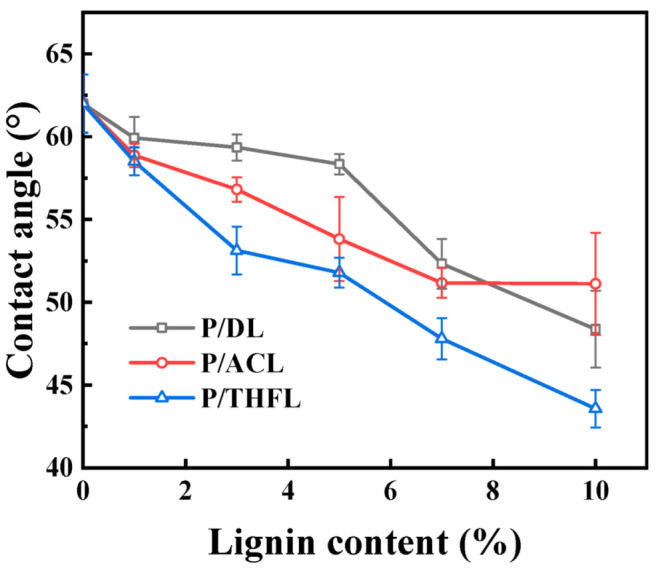
Contact angle of PBAT/lignin composite films.

The wetting properties of the film were evaluated by measuring the contact angle, with the results presented in Figure 4. Generally, the addition of lignin to the PBAT matrix reduces the contact angle and enhances hydrophilicity. This is mainly attributed to the presence of hydrophilic groups, such as hydroxyl, carboxyl, and methoxy groups. For all three PBAT/lignin composite films, the contact angle decreases and hydrophilicity increases with increasing lignin content. Acetone extensively dissolves hydrophilic lignin, so acetone-fractionated lignin exhibits moderate hydrophilicity; THF can dissolve most of the DL components, including the hydrophobic components in the sample [30]. The reason P/THFL is more hydrophilic than P/ACL is that in the casting process using THF as a solvent, THFL interacts more strongly with the solvent. When the solvent evaporates, THFL molecules may form a higher concentration tendency on the material surface due to differences in compatibility with the PBAT matrix, and the enhanced surface concentration of lignin increases the hydrophilicity of the material.

### 3.4. Mechanical Properties of PBAT/Lignin Composite Films

**Figure 5:**
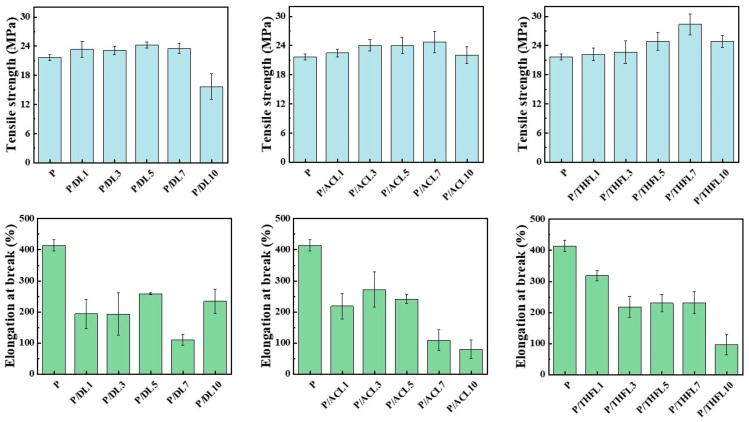
Tensile strength and elongation at break of PBAT/lignin composite films.

**Figure 6:**
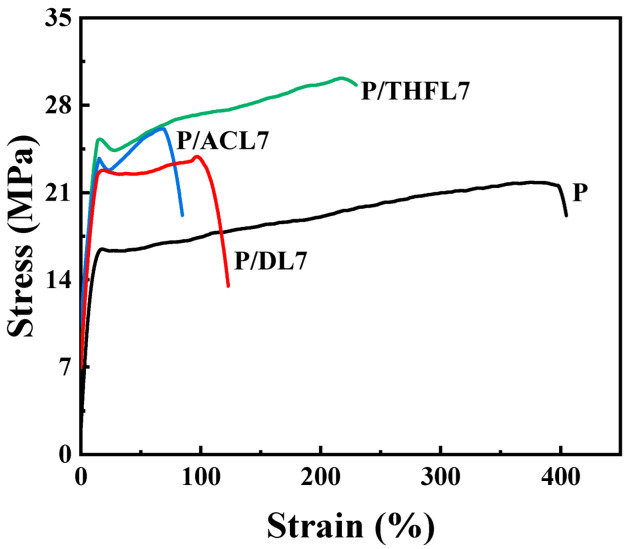
Typical stress–strain curves of PBAT/lignin composite films.

The influence of lignin fractionation on the mechanical properties of the composite films was evaluated by tensile testing, with the tensile strength and elongation at break presented in Figure 5 and Figure 6. As a general trend, the incorporation of lignin enhanced the tensile strength. For DL, the most significant improvement in mechanical properties was achieved at 5 wt% loading, demonstrating a 13.27% increase in tensile strength. However, further increasing the lignin content led to a decline in strength, which can be attributed to severe agglomeration and stress concentration, particularly at 10 wt% loading. This observation is consistent with previous work by Kargarzadeh et al. [11] demonstrating similar reinforcement effects when blending kraft lignin directly with PBAT. The mechanical enhancement mechanism can be attributed to lignin’s rigid aromatic structure functioning as an effective reinforcing filler at optimal loadings. Furthermore, the increased hydrophilicity of the composites suggests that lignin’s abundant hydroxyl groups provide additional hydrogen bonding sites [21], potentially forming interfacial bonds with PBAT’s carbonyl groups (RCOOR), thereby strengthening the matrix–filler interactions. Nevertheless, excessive lignin loading beyond the optimal concentration results in inadequate interfacial adhesion and compromised stress transfer, ultimately leading to reduced tensile performance.

For the P/ACL series, the tensile strength initially increased with a higher loading of ACL, reaching optimal performance at 7 wt% loading. The P/ACL7 composite exhibited tensile strength and elongation at break values of 24.69 MPa and 108.92%, respectively, which were 13.88% higher than the tensile strength of the neat PBAT film. The mechanical properties of the composite film prepared with lignin fractionated by acetone are superior to those of the composite film made with unfractionated lignin. This is mainly due to the interaction between the acetone-fractionated lignin and PBAT molecules, which enhances the dispersion of lignin and reduces its aggregation behavior. Similarly, the incorporation of THFL also enhanced the film’s tensile strength, with the maximum strength of 28.4 MPa achieved at 7 wt% loading—a 31% increase relative to pure PBAT. This more significant reinforcement effect demonstrates that THFL achieves more uniform dispersion within the PBAT matrix compared to other lignin fractions.

In summary, the limited reinforcement effect of unfractionated lignin can be ascribed to its heterogeneous composition, including insoluble components, which leads to poor dispersion in the PBAT matrix. In contrast, the fractionation process yields more homogeneous lignin fractions that can be more uniformly dispersed. In particular, the lignin fractionated by tetrahydrofuran has a more uniform property after fractionation and has a more stable interaction with PBAT. P/THF7 exhibits the optimal mechanical properties. Therefore, the composite films with 7 wt% lignin were selected for DSC and SEM characterization and compared with neat PBAT film to further elucidate the mechanism behind the improved mechanical properties.

### 3.5. Thermal Properties Analysis of PBAT/Lignin Composite Films

**Figure 7:**
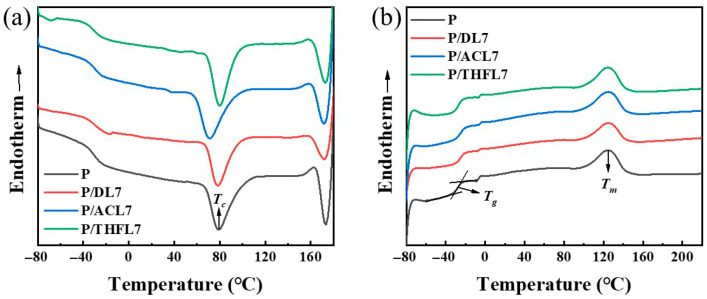
Cooling curves (**a**) and second warming curves (**b**) for P, P/DL7, P/ACL7, and P/THFL7.

Differential scanning calorimetry (DSC) was employed to investigate the thermal properties of the composite films and to further probe the compatibility between lignin and PBAT [21]. The glass transition temperature (*T_g_*), melting temperature (*T_m_*), and crystallization temperature (*T_c_*) of pure PBAT and composite films containing 7 wt% of DL, ACL, and THFL were determined. The results are summarized in Figure 7 and Table 2. As can be seen from Table 2, the *T_g_* of pure PBAT was found to be −27.5 °C, and the melting point *T_m_* was 124.5 °C, confirming its rubbery state at RT. After different lignins were added to the PBAT matrix, it can be observed that the *T_g_* values of P/DL and P/ACL both increased, indicating that the temperature at which the molecular chain segments begin to move freely has increased. The reason for the above phenomenon is that the hydrogen bonds and conjugation between DL, ACL, and PBAT molecules restrict the movement of PBAT molecular chains [31]. In addition to causing an increase in the glass transition temperature, this interaction will also be macroscopically manifested as an increase in the tensile strength of the film material.

Crystallinity is another crucial factor influencing mechanical properties, as a more ordered crystalline structure enhances intermolecular forces and improves stress transfer, thereby increasing tensile strength [32]. As shown in Table 2, the crystallinity of P/THFL7 is significantly higher than that of P, P/DL7, and P/ACL7. Concurrently, the crystallization temperature also increases, indicating that THFL acts as a nucleating agent and promotes the crystallization of PBAT chain segments. Considering the influence of the glass transition temperature, with the addition of 7 wt% lignin, the combined effect trend of the *T_g_* and *T_c_* of the composite film is consistent with the change in the mechanical properties of the film. In addition, the addition of lignin fractions has little effect on the melting temperature of the composite material.

### 3.6. Microstructural Morphology of PBAT/Lignin Composite Films

**Figure 8:**
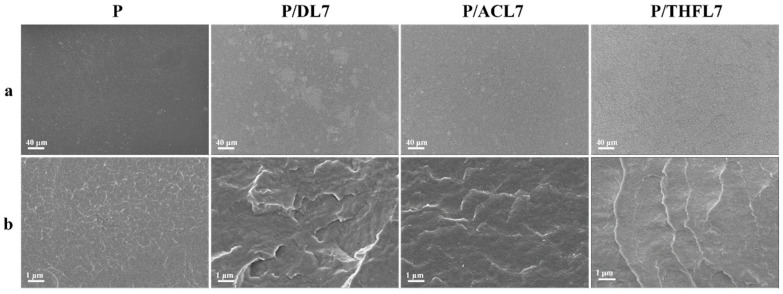
SEM images of PBAT/lignin composite films: surface (**a**), scale bar: 40 μm; cross-section, scale bar: 1 μm (**b**).

To visually assess the dispersion of lignin and the interfacial compatibility with PBAT, the micromorphology of the composite films was observed using scanning electron microscopy (SEM). The surface and cross-section images are presented in Figure 8. In the surface image of the composite film magnified 250 times (Figure 7a), it can be observed that the surface of pure PBAT appears relatively uniform and smooth. In the surface images of PBAT/lignin composite films, it can be seen that there are large agglomerates of DL in PBAT; ACL is distributed relatively uniformly, but some small agglomerates can still be seen; and THFL is uniformly dispersed in the PBAT matrix without agglomeration. The above results indicate that the compatibility of DL, ACL, and THFL increases in sequence, which is related to the enhanced conjugation and hydrogen bonding interactions in P/ACL and P/THFL analyzed by infrared spectroscopy. During the fractionation process, some impurities in DL and components that are not conducive to compatibility with PBAT are removed, making the composition of the fractionated lignin more uniform. This is beneficial to the interaction between lignin and PBAT, reduces problems such as phase separation caused by large differences in structure and polarity, and, thus, improves the compatibility.

The cryo-fractured cross-section (Figure 8b) provides further insights into the interfacial adhesion. The neat PBAT film exhibits a relatively smooth fracture surface with features characteristic of ductile fracture. In P/DL7, there are many white veins similar to “ridges” with varying lengths, which are longer and more prominent than those in pure PBAT. This indicates that there are relatively strong interactions inside the material, but the interactions are not uniform. In P/ACL7, the “ridges” become uniform and dispersed, which shows that the interaction between the interface of ACL and the PBAT matrix is enhanced. Therefore, stress can be transmitted more uniformly throughout the material system, avoiding the failure of the material caused by local stress concentration [33]. The “ridges” on the cross-section of P/THFL7 are further extended, spreading over the entire cross-section, and the overall structure is relatively dense. This is related to the increased crystallinity of P/THFL7. The densification of the internal structure of the material and the optimization of the microphase morphology together enhance the tensile strength of P/THFL7.

## 4. Conclusions

In this study, lignin was fractionated using organic solvents to reduce its heterogeneity and subsequently incorporated into a PBAT matrix by solvent casting. There is a significant conjugate effect and hydrogen bonding interaction between PBAT and lignin. The introduction of lignin reduces the contact angle of the composite material, with the series of composite films containing THFL showing the most significant changes. The addition of untreated lignin (DL) and acetone-soluble lignin fractions increases the glass transition temperature of the composite film and enhances its mechanical properties, with optimal results achieved at addition levels of 5 wt% and 7 wt%. Tetrahydrofuran-soluble lignin fractions can be uniformly dispersed in PBAT and act as nucleating agents to promote crystallization. At 7 wt% addition, the maximum tensile strength (28.4 MPa) was achieved, with a dense and uniform cross-section. This study facilitates the precise application of lignin. Without modifying lignin or adding functional additives, the simple and convenient method of lignin fractionation using organic solvents improves the compatibility between lignin and PBAT, thereby enhancing the mechanical and thermal properties of the composite film. The composite film prepared by solvent casting using tetrahydrofuran-soluble lignin and PBAT had significant advantages.

## Figures and Tables

**Table 1:** Yield and relative molecular mass of different fractions of lignin.

Sample	*Mn*/g·mol^−1^	*Mw*/g·mol^−1^	PDI	Yield/%
DL	1995	6759	3.39	\
ETL	1257	2187	1.74	31.70% ± 0.16%
ACL	1825	3657	2.00	14.64% ± 0.04%
THFL	3884	7050	1.82	19.77% ± 0.04%
RL	2885	8810	3.05	35.70% ± 0.29%

**Table 2:** DSC results for P, P/DL7, P/ACL7, and P/THFL7.

Composite Film	*T_g_*/°C	*T_m_*/°C	*T_c_*/°C	Δ*H_m_*/J·g^−1^	χ/%
P	−27.5	124.5	79.1	12.28	10.77
P/DL7	−25.5	124.9	78.3	11.5	10.85
P/ACL7	−25.6	125	71.1	11.33	10.69
P/THFL7	−27.5	124.7	79.9	14.3	13.49

*T_g_*: glass transition temperature; *T_m_*: melting temperature; *T_c_*: crystallization temperature; Δ*H_m_*: enthalpy of fusion; χ: crystallinity.

## Data Availability

Data will be made available on request.
